# Diet-Related Risk Factors for Chronic Noncommunicable Diseases in Italian Prisoners: B.A.C.I. (*Benessere All’interno delle Carceri Italiane*, Well-Being Inside the Italian Prisons) Project by the Italian Society of Penitentiary Medicine and Public Health (*S.I.M.S.Pe. Società Italiana di Medicina e Sanità Penitenziaria*)

**DOI:** 10.1007/s13668-023-00502-y

**Published:** 2023-11-10

**Authors:** Ludovica Verde, Antonio Maria Pagano, Monica de Leo, Claudia Vetrani, Antinea Ambretti, Luciano Lucania, Sergio Babudieri, Anna De Chiara, Annamaria Colao, Michele Corsi, Giovanna Muscogiuri, Luigi Barrea

**Affiliations:** 1 Department of Public Health, University of Naples Federico II, Via Sergio Pansini 5, 80131 Naples, Italy; 2 President S.I.M.S.Pe. Società Italiana Di Medicina E Sanità Penitenziaria (Italian Society of Penitentiary Medicine and Healthcare), Viale Bruno Buozzi 109, 00197 Rome, Italy; 3 Dipartimento Delle Attività Territoriali, ASL Salerno, U.O. Tutela Salute Adulti E Minori, Area Penale, 84124 Salerno, Italy; 4 Dipartimento Di Scienze Umanistiche, Università Telematica Pegaso, Centro Direzionale Isola F2, Via Porzio, 80143 Naples, Italy; 5 Specialista Ambulatoriale in Chirurgia, Responsabile, Giuseppe Panzera, Street Carcere Nuovo, 15 Istituto Penitenziario Di Reggio Calabria, 89100 Reggio Calabria, Italy; 6 Director S.I.M.S.Pe. Società Italiana di Medicina e Sanità Penitenziaria (Italian Society of Penitentiary Medicine and Healthcare). Viale Bruno Buozzi 109, 00197 Rome, Italy; 7 Unit of Infectious and Tropical Diseases, Department of Medicine, Surgery and Pharmacy, University of Sassari, 07100 Sassari, Italy; 8 Scientific Director S.I.M.S.Pe. Società Italiana di Medicina e Sanità Penitenziaria (Italian Society of Penitentiary Medicine and Healthcare). Viale Bruno Buozzi 109, 00197 Rome, Italy; 9 Unità di Endocrinologia, Diabetologia e Andrologia, Dipartimento di Medicina Clinica e Chirurgia, Università degli Studi di Napoli Federico II, Via Sergio Pansini 5, 80131 Naples, Italy; 10 Cattedra Unesco “Educazione Alla Salute E Allo Sviluppo Sostenibile”, University Federico II, 80131 Naples, Italy

**Keywords:** Prison, Inmates, Lifestyle, Noncommunicable diseases, Diet, Nutrition, Physical activity, Body composition

## Abstract

**Purpose of Review:**

The review aims to present an overview of inmate health, focusing on lifestyle-related diseases, physical activity levels, and nutritional status. It also presents the B.A.C.I. (*Benessere All’interno delle Carceri Italiane*, well-being inside the Italian prisons) project, which aims to offers an innovative path of prevention, diagnosis, and treatment of noncommunicable diseases (NCDs) related to unhealthy lifestyles in prisons in the Campania region, Italy.

**Recent Findings:**

The global prison population has risen by 24% since the year 2000, with over 10.77 million people detained worldwide in 2021. In Italy alone, there are currently over 57,000 inmates. Inmates face a higher risk of NCDs such as cardiovascular disease due to unhealthy lifestyles characterized by poor diets and lack of physical activity. Additionally, sleep disorders, particularly insomnia, are prevalent among inmates, further contributing to health disparities. While physical activity has shown positive effects on inmate well-being, there is limited research on nutritional status and interventions in prison populations.

**Summary:**

Providing quality healthcare to inmates is an international policy norm, but the standards vary globally and are often inadequate. The economic burden of NCDs is rising, and this is exacerbated in prisons, making it challenging for individuals to reintegrate into society after release.

## Introduction

Global prison population has increased by 24% since around the year 2000, a growth rate slightly lower than the estimated increase in the world’s overall population during that time (28%) [1]. According to the Italian Justice Department, in Italy, there currently are over 57,000 inmates [2]. However, there is significant variation in prison population growth both between and within continents. In 2021, the global count of persons incarcerated in penal institutions will exceed 10.77 million, as reported by the World Prison Population List [1]. In addition, data from the US National Corrections Reporting Programme revealed a steady increase in the proportion of elderly inmates (aged 55 years and older) and female inmates, outnumbering young adults aged 18 to 24 [3]. This expanding and diverse prison population underlines the importance of addressing health in prisons as a vital aspect of public health. It also highlights the crucial role of prisons in mitigating health inequalities.

Inmates carry a significant load of physical and mental health conditions in contrast to the broader populace. This discrepancy in health has been linked to a range of behavioral and socioeconomic elements [4], encompassing factors like unhealthy eating habits and insufficient physical exercise [5]. It is worth mentioning that inmates face a higher risk of noncommunicable disease (NCDs) such as cardiovascular disease compared to the general population [6]. Distinct lifestyles are prevalent among the prison population, characterized by diets rich in sugar and saturated fat while lacking in protein, a lack of physical activity, smoking habits, and increased consumption of alcohol and drugs [4]. These behaviors significantly increase the likelihood of developing NCDs, which are further exacerbated during imprisonment [4]. Furthermore, several studies have shown that, during detention, inmates are more likely to develop sleep disorders in general, or more specifically, insomnia [7, 8]. Of note, there is a bidirectional relationship between sleep disorders, poor eating habits, and increased risk of NCDs, implying that each of these factors can negatively influence the others, creating a vicious circle that can lead to serious health consequences [9].

While there is some evidence suggesting the positive impact of physical activity levels on the well-being and quality of life of inmates, particularly in relation to sports education programs which have been shown to benefit inmates in terms of social development, reduction of cardiovascular and metabolic diseases, and cognitive decline [10–12], there is considerably less research on the nutritional status, body composition, and nutritional interventions among inmates. It remains unclear whether a healthy lifestyle, i.e., a balanced diet and proper physical activity, under the guidance of a qualified nutritionist can effectively improve health in prison population.

It has become a universally accepted policy standard that correctional institutions should offer medical services at a level equivalent to that available within the broader community [13]. Nonetheless, the quality of healthcare in prisons is thought to differ globally, partly due to resource constraints; even in wealthier nations, there have been reports of inconsistent or insufficient provision [14]. In this context, according to Sridhar et al., only a few countries provide information on the expenses related to healthcare services in prisons. When such data is presented, there exists a lack of transparency and consistency in terms of what expenses are encompassed [15].

Therefore, as the prison population represents a significant percentage of the population suffering from NCDs, in addition to the increased health care costs for prison authorities, individuals who have developed this type of diseases while in prison may find it more difficult to reintroduce themselves into society after detention. Of note, there is evidence that increased spending on primary health care has the potential to reduce total health expenditure [16], and for this reason, it is of urgent importance to assess gaps and appropriate interventions also in prison population. Specifically, in Italian prisons, especially in those regions where the prevalence of overweight, obesity, and NCDs is high, there is a growing need for specific prevention and/or treatment campaigns of NCDs related to unhealthy lifestyles in order to improve the knowledge and awareness of this disease among the prison population.

The purpose of this review was (1) to provide an overview of the health status of inmates, with a focus on lifestyle-related diseases (such as NCDs and sleep disorders); (2) to summarize the current evidence on the physical activity levels, nutritional status, and body composition of inmates; and (3) to report on the current evidence on lifestyle interventions (physical activity programmes and nutritional interventions, if any) conducted in prison population to date. As a last aim, this review presents the B.A.C.I. (*Benessere All’interno delle Carceri Italiane*, well-being inside the Italian prisons) a project sponsored by the 5 × 1000 funds of the Pegaso Telematic University of prevention, diagnosis, and treatment of NCDs related to unhealthy lifestyles held in prisons in the Campania region, Italy. This project offers an innovative path, which could easily be implemented in all prisons in order to prevent NCDs and treat them early through the improvement of lifestyles so as to reduce the development of pathologies and thus reducing public health expenditure.

## Health Status of Prison Population

### Noncommunicable Diseases (NCDs)

NCDs, encompassing conditions like cardiovascular diseases, cancer, type 2 diabetes, and chronic respiratory disorders, stand as the foremost global causes of mortality, accounting for 74% of all worldwide deaths [17]. These NCDs share pivotal modifiable behavioral risk factors, such as tobacco consumption, unhealthy dietary habits, insufficient physical activity, and the detrimental use of alcohol, ultimately resulting in issues like overweight and obesity, along with changes in the cardiometabolic profile. NCDs remain a significant public health hurdle across all nations, including low- and middle-income countries where over three quarters of NCD-related fatalities occur [17].

Although NCDs affect people of all nationalities, ages, and classes, there are unequivocal inequalities in the burden of these diseases between individuals in vulnerable situations (e.g., in prisons or detention) and the general population [18]. According to a survey conducted in the USA, inmates exhibited elevated age-adjusted rates of conditions such as hypertension, type 2 diabetes, asthma, and arthritis in comparison to the broader populace [19].

In particular, cardiovascular diseases are a leading cause of death among inmates, and they often contribute to mortality immediately following release from correctional facilities [20]. Of note, research indicates that there is a link between a history of incarceration and cardiovascular risk factors, morbidity, and cardiovascular disease-related mortality [21–23]. However, accurately determining the causal effects of incarceration is complicated by the presence of multiple confounding factors, such as life course and other social factors that are associated with being incarcerated. A meta-regression (26 studies included) aimed to quantify the relationship between incarceration and trends in major cardiovascular disease among current of former people in prison found out only a trend towards rising blood pressure or prevalence of hypertension during incarceration and an increased incidence of hypertension following incarceration [24].

Type 2 diabetes is an ever-increasing public health challenge and this can also be expected in prison population [25]. A cross-sectional study among 437 inmates from the Yaoundé Central Prison found out a prevalence of type 2 diabetes of 9.4% while the national prevalence was of 5.8% [26]. Among this cohort of inmates, the primary contributors to cardiovascular risk were sedentariness (91.1%), hypertension (39.6%), smoking (31.6%), and alcohol use (28.1%) [26]. Regrettably, despite the presence of guidelines for managing type 2 diabetes within correctional facilities [27, 28], the provision of such care varies widely and is frequently inadequate [25]. The utilization of external contractors and constrained budgets incentivizes cost reduction measures that can jeopardize the well-being of individuals with type 2 diabetes and other inmates. This is evident through subpar food from contracted sources, designed to minimize expenses [25]. Suboptimal treatment of type 2 diabetes, which is also partly determined by unhealthy diet and physical inactivity, can lead to poor glycemic control and increase the risk of health complications in inmates [25].

Contact with the criminal justice system should represent a public health opportunity to prevent and improve these clinical conditions in inmates. The prison regime represents an important venue in reducing an individual’s exposure to risk factors for NCDs given the social context in which inmates must live for several years. Prisons, therefore, have a responsibility to create a healthy environment and engage in individual health promotion, throughout also promotion of a healthy lifestyle.

### Sleep Disorders in Prison Population

From a sleep quality point of view, inmates are a vulnerable group, who have a high risk of sleep disorders [8, 29, 30]. Very recently, Sheppard and. Hogan conducted a systematic review in which they identified 12 cross-sectional studies that reported on the prevalence of insomnia or poor sleep quality using in-prison self-report measures among adult inmates [31]. The results showed that sleep quality within prisons was deteriorated significantly in recent years and that the estimated prevalence rates of sleep disorders in prisons were variable. The prevalence of poor sleep quality and insomnia in the prison population was variable but still higher than the average for the general population. According to authors, the prevalence of poor sleep quality among inmates ranged from 26.9 to 72.5%, exceeding the 25 to 36% range observed in the general population. Similarly, the prevalence of insomnia among inmates ranged from 25 to 36%, surpassing the 6 to 30% range seen in the general population [31]. Similarly, Dewa et al. conducted an integrative review examining the prevalence and management of insomnia in prisons [30]. The authors concluded that sleep disorders are common and disabling among inmates, are linked to comorbid conditions, and are negatively affected by the prison environment, which usually provides limited scope for effective management [30].

Within prisons, psychosocial and sociocultural factors may contribute to sleep challenges [32, 33]. For example, incompatible sleep behavior has been reported within prisons, where beds become places to sit, watch TV, and eat meals, but not just to sleep [33]. Environmental elements such as noise, limited physical activity, extreme temperatures, monotony, and other aspects associated with institutional life can also play a role in diminishing the amount of sleep [34]. In addition, inmates are known to have a high incidence of mental disorders, which consequently increases the risk of sleep disorders [30].

Recognizing and effectively addressing sleep disorders among inmates are crucial. This is vital due to the wide-ranging effects of these disorders, which encompass cognitive impairments, heightened aggression, reduced impulse control, disturbances in emotional regulation, and an elevated accident risk [33], as well as the potential development of cardiometabolic diseases [35]. In this regard, a recent study explored the efficacy of a one-session cognitive behavioral therapy for insomnia (CBT-I) and a self-management pamphlet for acute insomnia in 30 adult male inmates [36]. Results demonstrated significant reductions in insomnia severity and notable improvements in mood symptoms [36].

Research has shown that sleep disorders are associated with increased consumption of energy and fat, alongside a potential decrease in fruit consumption and adoption of unhealthy dietary habits [10]. Moreover, individuals with insufficient sleep might display irregular eating routines, including fewer main meals and a higher frequency of energy-rich snacks during nighttime. Although the influence of inadequate sleep on dietary habits is generally modest, prolonged sleep deprivation can contribute to an increased susceptibility to obesity and related NCDs. The mechanisms underlying the relationships between sleep duration and dietary choices are intricate and involve factors like appetite-regulating hormones, pleasure-related pathways, extended eating periods, and changes in timing of meals [10].

Given the evidence for causal relationships between sleep disorders, eating habits, and cardiometabolic health, health promotion strategies should prioritize improved sleep as an additional factor in health and weight management, particularly in prison population.

Sleep quality can be easily assessed with standardized indirect measurements designed to identify symptoms of insomnia and sleep disorders, such as the Pittsburgh Sleep Quality Index (PSQI) questionnaire [37]. This method of assessing sleep quality is widely applied in scientific literature, administered by qualified nutritionists in various studies [38–40]. Thus, a qualified nutritionist could be of additional value in assessing the sleep quality of inmates, in order to better identify those at greater risk of health complications.

## Physical Activity Levels, Nutritional Status, and Body Composition in Prison Population

### Physical Activity Levels in Prison Population

Physical activity plays an important role as a protective factor against NCDs [41] and for this reason, it should be considered in all prison populations to improve health conditions at no cost. According to the WHO, in the 18–64 age group, it is recommended to perform at least 150 to 300 min of moderate-intensity aerobic physical activity per week, or at least 75 to 150 min of intense aerobic physical activity [42].

The available scientific literature presents a significant variability in the reported levels of physical activity or sedentary behaviors among inmates. In a study by Cashin et al., involving 914 inmates, it was observed that 87% of men and 73% of women mentioned engaging in exercise within a 4-week span [43]. The average weekly exercise duration was calculated at 73.2 (± 85.5, ranging from 0 to 621.4) minutes. Moreover, only 66% of men and 51% of women in the same study reported daily physical activity [43]. Comparable low levels of physical activity were identified in a group of 505 women inmates [44]. Remarkably, merely 13.1% of these individuals participated in 30 min or more of moderate activity at least five times weekly [44]. Camplain et al. also reported findings indicating that over half (56%) of 82 women observed during recreation periods (a designated time for physical activity) were sedentary, while 4% were involved in intense physical activity, and roughly 40% engaged in walking or other similarly moderate physical activities [45].

Overall, current data suggest that physical activity levels among inmates are generally low and that there is a need for interventions to increase physical activity in this population to improve their health outcomes.

### Nutritional Status and Body Composition in Prison Population

Malnutrition among individuals who are either underweight or overweight presents a significant cause of both mortality and morbidity in prisons across both developed and developing nations [46]. In developed countries, inmates are experiencing more rapid weight gain compared to the general population [47]. Research has indicated that individuals serving lengthy prison sentences tend to gain weight, leading to increased obesity rates during their incarceration [47]. Moreover, there is a noticeable increase in obesity-related health issues, including type 2 diabetes, hypertension, and atherosclerosis, among inmates [26, 48, 49]. Conversely, underweight conditions are also prevalent among inmates, particularly in developing countries [50].

Herbert et al. conducted a systematic review of the literature which suggests that the composition of inmates’ diets was in part responsible for the increased risk of NCDs in prison population [19]. In fact, diets in prisons were high in refined carbohydrates and saturated fats and very poor in fiber, vitamins, and minerals due to the scarcity of fruits and vegetables. The problem of these unhealthy dietary regimens could be further aggravated by the fact that inmates may purchase extra snacks, which tend to be energy-dense, rich in salt which leads to depletion of vitamin and mineral salt reserves, worsening the nutritional picture and predisposing these individuals to cardiovascular, metabolic, and oncological diseases with major repercussions on health care spending [19]. Of note, other studies investigating community food services in prisons reported that special dietary needs (type 2 diabetes, dyslipidaemia, etc.) could not be met both because of budget problems but mainly because of the lack of qualified personnel (Nutritionist) capable of drawing up balanced and low-cost dietary plans [51, 52]. Plugge et al. reported that inadequate nutrition and lack of exercise put inmates at serious risk of developing cardiovascular disease, leading to increased health care costs that could be easily avoided through proper nutrition and individualized exercise programs [44].

Obesity, a major contributor to the global burden of NCDs, spreads in prisons in a similar way to the general population [53]. The prevalent approach for evaluating obesity is through the BMI, yet numerous research studies suggest that the distribution of central fat and the proportionate decline in fat-free mass could potentially carry greater significance than BMI itself when determining the health risks linked to obesity.

For this reason, it is important to evaluate body composition to allow more accurate assessment of an individual’s health and well-being and to determine personalized strategies to improve overall health and well-being. Unfortunately, the majority of the studies in inmates are limited to the evaluation of BMI to assess overweight and obesity [54–56]. To our knowledge, to date, there are no studies that have specifically assessed body composition in the prison population.

## Lifestyle Interventions in Prison Population

### Physical Activity Interventions in Prison Population

Several scientific evidences have reported the beneficial role of physical activity carried out through individualized programs prescribed by qualified personnel, on cardiometabolic health in inmates.

Battaglia et al. conducted an estimation of the type of physical activity that could enhance the health condition and fitness levels of inmates [11]. They assessed the effectiveness of a 9-month intervention program involving physical activity on the psychological well-being of 64 inmates. The participants were grouped randomly into three categories: resistance training, high-intensity strength training, and no exercise. The researchers found that both forms of exercise (resistance training and strength training) led to notable reductions in depression scale scores when compared to the control group, where the average depression scale scores increased. The study’s outcome was that the physical activity program had a positive impact on inmates’ mood, anxiety levels, and overall mental health [11]. Similarly, Mannocci et al. undertook a multicenter cross-sectional study to examine the relationship between physical activity and the quality of life among incarcerated individuals in Italy [12]. Inmates from eight prisons compiled a questionnaire (a total of 636 questionnaires were compiled). The authors found a positive association between quality of life and physical activity level. They also showed that years of incarceration and age were important aspects of the overall assessment of quality of life. Time spent on exercise was indeed positively correlated with the inmate’s age and years spent in prison. Inmates with long-term sentences and older people felt more of a need to organize activities and had interests in improving the way they spend their time and achieving better life satisfaction [12]. A double-case study by Amtmann and Kukay examined the effects of fitness coaching on two inmates in a juvenile detention facility in southwestern Montana [57]. After the 8-week program, both participants made improvements in physical fitness and both perceived positive effects on self-concept and general sense of well-being from participation in this program [57]. More recently, a study by Bueno-Antequera et al. investigated the impacts of a 12-week intervention involving a combination of moderate to high-intensity aerobic and strength exercises on 41 inmates with psychiatric disorders [58]. The researchers found that the exercise regimen led to notable enhancements in cardiorespiratory fitness, upper body strength, and anthropometric measurements [58]. In a systematic review conducted by Mohan et al., interventions aimed at improving factors or behaviors linked to cardiovascular health within incarcerated populations were examined [59]. The review concluded that supervised physical activity had a positive effect on determinants such as blood pressure and cardiovascular issues during the period of incarceration [59]. Additionally, Sanchez-Lastra et al. conducted a systematic review that evaluated the effectiveness of exercise programs implemented among inmates, demonstrating that exercise initiatives within correctional settings present a practical and beneficial approach for enhancing the physical and mental health of inmates [60]. In light of the reduction in depression scores, Battaglia et al. conclude that 1 h of moderate physical activity per week would be enough for inmates to improve mental health [60]. Finally, in a recent systematic review of the scientific literature, Papa et al. discussed the general health conditions and cardiovascular risk profile in inmates compared to the general population and evaluated whether or not exercise could be a useful tool for the prevention of these diseases in inmates [13]. Nine studies examined the health status of inmates, with five studies focusing on the occurrence of cardiovascular disease and coronary heart disease within the prison population. Additionally, ten studies assessed the practicality and effectiveness of exercise initiatives among inmates. Inmates can derive advantages from sports education programs. It appears that engagement in supervised exercise led by qualified professionals emerges as the most efficacious approach to address the challenges of imprisonment. Furthermore, it appears that sports programs hold promise as a valuable strategy to enhance the physical and mental well-being of inmates, alongside diminishing risk factors associated with cardiovascular health [13].

Although the beneficial effects of physical activity on cardiometabolic health are well-recognized, still little is being done in prison population to incentivize them to more active behaviors in order to improve the quality of life in inmates and reduce the development of NCDs related to unhealthy lifestyles. In this context, the qualified nutritionist would represent the clinical professional figure who can improve these components leading to the reduction of health care expenditures related to drug therapy costs for cardiometabolic diseases.

### Nutritional Interventions in Prison Population

There are few studies that have examined the results of nutritional interventions within inmates. Gil-Delgado et al. investigated the impact of participating in a nutritional program involving health education and the availability of a balanced diet on cardiovascular risk and metabolic syndrome indicators [61]. The researchers conducted a nonrandomized prospective cohort study spanning a year in a Spanish prison. Anthropometric and blood biochemical factors were assessed at various intervals throughout the year to analyze the effects of dietary changes on 139 inmates, of which 95 successfully completed the program. The study revealed that 86.3% of the participants adjusted their diets. Substantial enhancements were observed in weight, fat mass, abdominal circumference, and diastolic blood pressure, contributing to an overall reduction in cardiovascular risk [61]. Similarly, Sioen et al. explored the impact of providing a diet enriched in alpha-linolenic acid (around 5 g/day compared to the standard 2.8 g/day) on cardiovascular risk factors within a group of 59 healthy male inmates in Belgium [62]. Their single-blind field trial with pre- and postmeasurements showed that diastolic blood pressure significantly decreased, while the HDL cholesterol level increased in nonsmoking participants. Although waist circumference, weight, BMI, and systolic blood pressure remained unchanged, the results suggest that healthier diets can yield positive outcomes in prison environments, given the availability of appropriate financial and human resources to provide nutritious food options [62]. Finally, a study conducted by Loeb and Steffensmeier delved into the obstacles to health promotion faced by inmates aged 50 and above [63]. Focus groups involving 42 inmates illuminated that concerns related to food were among the factors impeding prisoners’ capacity to embrace a healthy lifestyle. Inmates indicated their attempts at adopting self-care strategies, which encompassed elements like maintaining a positive outlook, managing their diet and weight, participating in physical activities, and safeguarding their well-being [63]. Interestingly, a Danish study underscored the advantages of allowing inmates to prepare their own meals and decide what to eat [64]. The overall findings indicated that meal-related contemplation and preparation occupy inmates’ time, with the system receiving positive feedback as it allows them to connect with their responsibility for making health-conscious choices [64].

It is also interesting to note that a Dutch case–control study reported the reduction of antisocial behaviors among inmates who received dietary supplements (vitamins, minerals, and essential fatty acids) compared to placebo, probably due to proper supplementation of electrolytes, minerals, and vitamins that evidently were poorly supplied in the diet given the reduced intake of fruits and vegetables that are the main source of micronutrients [65]. It should also be noted that the results of the available scientific literature indicate the key role played by proper nutrition in both improving the cardiometabolic health of inmates and reducing their aggressive behaviors [42, 65], providing a compelling reason to include a qualified nutritionist in the prison staff in order to improve diet quality. This could include simple and low-cost measures. First, food could be provided as an appetizing menu, and monotonous menus could be avoided. For example, this could be done by ensuring that rice or pasta is not provided every day of the week (since the high-glycemic index carbohydrate load is associated with overweight and cardiometabolic disease [66]), but could be replaced by other grains, such as spelt and/or barley, which are lower cost but nutritionally better. Second, while having a menu program that is repeated every one or 2 weeks causes boredom and food monotony after a few months, thus, many inmates can accommodate even small changes in menus each week and even surprise meals.

### Assessment of Nutritional Status and Body Composition

Accurate and valid body composition assessment holds significance for diagnosing nutritional status, pinpointing pertinent indicators of progress, and gauging the effectiveness of existing and upcoming nutritional interventions [67–69]. Consequently, body composition assessment constitutes a crucial component of evaluating nutritional well-being, offering valuable prognostic insights and a means to track the impact of nutrition and nutrition-based interventions on the advancement of diseases. Parameters considered for nutritional status evaluation encompass height, weight, and BMI, as well as alternative measures like body circumferences [67].

Very important is the identification and to the evaluation of sarcopenia, originally defined as the age-related loss of muscle mass now identified as the dysfunction of three parameters: muscle mass, muscle strength, and physical performance. There is a growing acknowledgment that muscle strength is not solely governed by muscle mass; multiple other factors come into play. These factors encompass neural aspects, muscle function-related factors like underlying diseases (e.g., myopathy, neuropathy), intramuscular adipose tissue, inflammation, hypoxia, peripheral vascular disease, oxidative stress, electrolyte imbalance, and inactivity [67]. Furthermore, conditions like vitamin D or iron deficiency and disruptions in fat and glucose metabolism can also impact muscle function. Given this enhanced comprehension, the term “dinapenia” has been introduced to distinguish between diminished muscle mass and reduced muscle strength. Moreover, it is increasingly understood that muscle function might hold more significance than sheer mass when it comes to the risk of disability and mortality. Consequently, the emphasis in outcomes has shifted towards targeting enhancements in muscle strength through novel nutritional and/or exercise interventions, rather than exclusively concentrating on changes in muscle mass [67].

Considering this advanced insight, the assessment and tracking of body composition should increasingly focus on techniques that exhibit impairment and improvement in muscle strength and function, serving as crucial early indicators of risk and mortality. Bioimpedance analysis (BIA) has been utilized in clinical practice for a considerable duration. It was initially devised to gauge body water, then expand to assess body composition, and ultimately to prognosticate disease severity and prognosis [67]. BIA is deemed secure, straightforward, minimally invasive, and relatively cost-effective and exhibits greater reproducibility (< 1%) compared to skinfold measurements. Its results are promptly accessible, and several portable devices are available, rendering it an attractive avenue for clinically evaluating body composition [67].

Monitoring muscle strength and physical performance, key aspects of sarcopenia, stands as vital for assessing body composition in response to nutritional interventions [67]. Grip dynamometry, one of the most frequently employed techniques to assess muscle strength, measures the grip strength of the upper extremity. It is a portable, uncomplicated, and affordable method that has demonstrated predictive capabilities for postoperative risk, hospitalization, disability, cardiovascular disease, and overall mortality, irrespective of muscle mass [67]. The gold standard, recommended by the American Society of Hand Therapists (ASHT), involves measuring grip strength in a seated position with specific criteria for hand positioning and averaging three maximal grip efforts from both dominant and nondominant hands. It is noteworthy that the choice of dynamometer can influence outcomes, with the Jamar dynamometer (Lafayette Instruments, Lafayette, IN, USA) commonly adopted as the gold standard [46].

Waist circumference serves as an indicator of central fat distribution and is typically easy to measure. It effectively predicts the risk of cardiometabolic health issues and mortality [70]. To ensure accuracy, a standardized procedure must be followed [71]. This involves having the individual remove any bulky or tight outer clothing and heeled shoes, empty their bladder, and stand upright with arms at their sides. The measuring tape is positioned around the body, midway between the iliac crest and the lower rib’s costal margin. It is crucial to maintain a horizontal and untwisted tape placement. The individual is instructed to look ahead and exhale, and the measurement is taken at the end of exhalation, repeating the process for accuracy. While various anatomical sites have been suggested for waist circumference measurement, such as the minimum abdominal circumference and the umbilical level, these yield notably distinct values. This variance could hinder consistent measurements in clinical settings, emphasizing the importance of documenting the site of measurement for practitioners [72].

In conclusion, nutrition status is the result of the assessment of body composition, dietary intake, and nutrient absorption and utilization. Assessment of nutrition status should always precede any dietary intervention, representing a diagnostic procedure, an integral part of nutritional semeiotics. However, to date, no study has evaluated body composition and its association with the incidence of disease within prisons.

## The B.A.C.I. Project

**Fig. 1:**
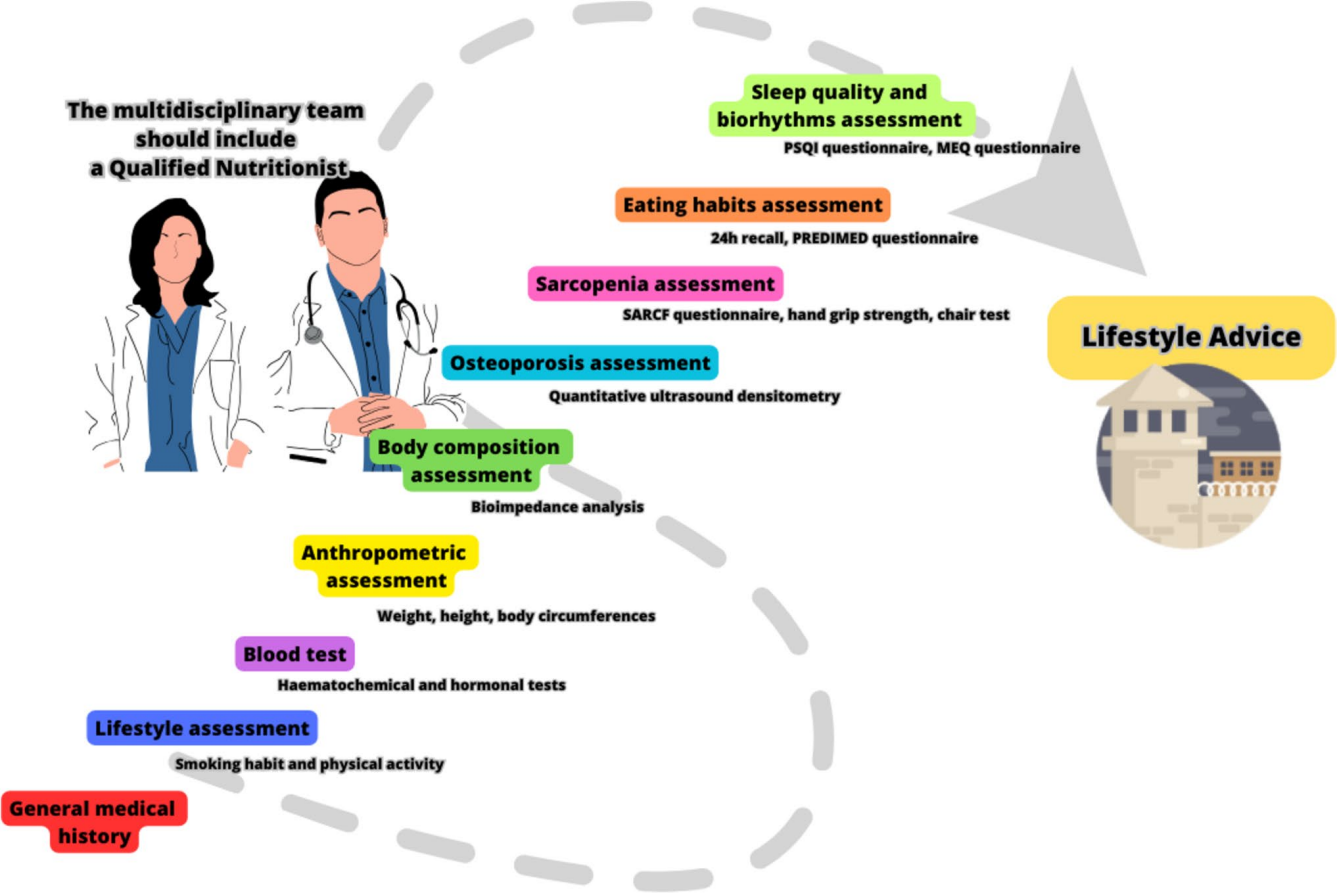
Health path for inmates. B.A.C.I. (*Benessere All'interno delle Carceri Italiane*) project, an innovative pathway for the prevention, diagnosis, and treatment of noncommunicable diseases linked to unhealthy lifestyles in the prisons of the Campania region, Italy. PSQI Pittsburgh Sleep Quality Index, MEQ Morningness–Eveningness Questionnaire

This project aims to transform prisons from punitive places to rehabilitative environments by improving the health and well-being of inmates through proper nutrition and physical activity. The project fills a serious health gap in the prison population by assessing the nutritional status of inmates, preventing the development of overweight and obesity, and reducing the incidence of lifestyle-associated diseases. The qualified nutritionist, within the multidisciplinary team, plays a vital role in promoting healthy eating and physical activity programs, which can improve the health status of inmates (Fig. 1).

The multidisciplinary team consisting of a physician, nurse, and nutritionist carries out different clinical evaluations during the first visit to prisons. The physician and nurse conduct a clinical evaluation to collect the family and personal clinical history of inmates, along with their medication use. This information is essential to identify any pre-existing medical conditions that may affect their health and well-being during incarceration. Blood pressure measurement and routine biochemistry tests are performed to assess the overall health status of inmates and to identify any potential risk factors for cardiovascular and metabolic diseases. The nutritionist evaluates the anthropometric parameters, including weight, height, and waist and hip circumference for a comprehensive anthropometric assessment. Bioelectrical impedance analysis is used to assess body composition, which can provide valuable information on the risk of developing cardiometabolic diseases. Physical activity is assessed using the IPAQ questionnaire, one of the most widely used validated instruments internationally for research and clinical use. Calcaneal heel ultrasound is performed to measure bone density using a quantitative ultrasound (QUS) bone densitometer [72]. This evaluation is essential to identify any potential risk of osteoporosis in inmates. The nutritionist also assesses the presence of sarcopenia, a condition characterized by the loss of muscle mass and strength, through questionnaires (SARC-F) and functional tests (chair test and handgrip strength test) [73]. This evaluation is essential to identify any potential risk of mobility problems in inmates. Questionnaires aimed at investigating eating habits (24h recall), adherence to Mediterranean diet (PREDIMED questionnaire [74]), and biorhythms (MEQ questionnaire [75]), along with quality of sleep (PSQI questionnaire [37]), are also conducted. These evaluations are essential to identify any potential risk of developing lifestyle-associated diseases and to provide lifestyle advice aimed at improving the alterations found. At the end of these evaluations, inmates are informed of the results on their state of health, and they are given lifestyle advice aimed at improving the alterations found.

This project contributes to the implementation of knowledge on health risk factors that predispose to NCDs in the prison population. In addition, data on diet adherence, measurement of body composition, bone and musculoskeletal health, sleep disorders and biorhythms, and cardiometabolic risk assessment are provided for the first time. These results are used to develop therapeutic strategies for improving health status and possibly reducing costs for the health care system and for managing the delivery of health care services in prisons.

## Conclusion

A healthy diet and proper physical activity are key components to maintaining a healthy lifestyle and preventing rising rates of NCDs, particularly obesity and cardiovascular diseases [76]. As previously reported, cardiovascular diseases continue to be the leading causes of mortality in both the general and prison populations, in the latter population most alarmingly [19, 20]. Despite this knowledge, choices that promote cardiometabolic health based on lifestyle modifications in prison population are very scarce; this is basically due to the lack of the figure of a qualified nutritionist to implement nutrition promotion programs among inmates. Furthermore, while there are health programs based on lifestyle modifications (diet and physical activity) in the general population, these programs are mainly absent in prison population. This is worrisome considering the increasing number of inmates [2] and the incidence of NCDs [19, 20], as well as the medical expenses for the treatment of these diseases in prison population [77]. In this context, ensuring a healthy diet and proper physical activity would represent feasible, very low-cost preventive measures to enable inmates to maintain or achieve good health status not only physically and clinically but also psychologically, given the benefits of proper nutrition also on mental health [78] and sleep disorders [79], which, as we have previously reported, are not only a social problem but also, and especially, a health problem [35]. Improving the health of prisoners not only is a matter of human rights but also has significant public health implications, given that most prisoners eventually return to society. Long periods of incarceration can lead to weight gain and increase the risk of NCDs, which can be costly to treat. Encouraging healthy diets and physical activity during incarceration can have positive effects on other health outcomes, such as mental health. To address this gap in the prison population, it is important to bring qualified nutritionists into this clinical setting to assess the nutritional status of prisoners and provide tools to improve their health, prevent overweight and obesity, and reduce the incidence of diseases associated with unhealthy lifestyles. A qualified nutritionist promoting healthy eating and physical activity programs could be an effective public health strategy to improve the health status of inmates, leading to increased well-being and decreased healthcare expenditure. Ultimately, this approach would benefit not only inmates but also the national healthcare system in the long run.
